# Supplementary material for: Are carbon emissions trading and green financial instruments synergistic? -Comprehensive quantitative research based on content analysis
Source: PLoS One. 2024 Mar 7;19(3):e0298601. doi: 10.1371/journal.pone.0298601 (PMC10919601; doi:10.1371/journal.pone.0298601)
Supplement: S1 Data — (ZIP) [file pone.0298601.s001.zip › Data/地级市Z_F工作报告.zip!/║┌┴·╜¡/╦τ╗»╩╨/└┤╫╘╣1⁄2╓┌║┼í╛┬φ┐╦╩2╛▌═°í┐_2010.docx]

各位代表：

现在，我代表市人民政府向大会报告工作，请审议，并请列席会议的同志提出意见。

一、2009年政府工作情况

2009年是极不寻常、极不平凡的一年，也是加快发展取得重大成就的一年。面对金融危机的冲击，我们坚决落实省委、省政府和市委的决策部署，以“两区”建设为牵动，保增长、保民生、保稳定，实现了经济社会持续较快发展，较好地完成了市二届四次人代会确定的各项工作任务。

全市实现国内生产总值606亿元，同比增长13.8%；全口径财政收入39.4亿元，增长15%，一般预算收入19.3亿元，增长21.8%；城镇固定资产投资232亿元，增长67.2%；社会消费品零售总额220.9亿元，增长18.7%，城镇居民人均可支配收入8133元，增长14.4%，农民人均纯收入4371元，增长16.1%。以上指标增长均高于全省平均增幅，并提前一年实现“十一五”规划目标。肇东继续保持全省十强之首，并在全国百强县排名中升至89位，安达继续稳居全省十强和全国中小城市“四个百强”，北林、望奎、明水财政收入三年翻一番还多，庆安一般预算收入增幅列全市之首达43.6%，其他县市发展步伐进一步加快。

——项目建设和固定资产投资成果丰硕。全社会固定资产投资近300亿元，是投资力度最大的一年。海伦、望奎、青冈、庆安、安达、北林、兰西城镇固定资产投资增幅均超过70%，市本级、肇东、安达、北林、望奎的投资增量都在10亿元以上。招商引资和项目建设势头强劲。全市新建续建3000万元以上项目202个，亿元以上项目76个，完成投资122.5亿元，占城镇固定资产投资的52.8%。新引进项目305个，其中亿元以上项目41个，到位资金86.5亿元，增长31.3%。市本级保利光伏材料、肇东光电产业园、安达贝因美二期、望奎三精新中药、庆安响水河矿开发二期、明水格林谷氨酸钠、海伦干法纸浆、北林生物质发电、青冈绿春源饲料、绥棱复合地板、兰西生物固化燃料等项目均是单体规模大、持续发展能力比较强的项目。安达MTBE和海伦九鼎酵母项目实现了当年引进、当年投产。基础设施建设全面大上。投资11.3亿元建设农村公路3357公里，是年初计划的2.5倍，总里程和投资额连续四年居全省首位。支持伊绥、绥北两条过境高速公路建设，落实动迁征地资金6亿元。城市基础建设投资持续增加。仅道路、给排水、绿化、亮化、广场及公园等建设完成投资10亿元，启动城市“三供两治”项目28个，总投资25亿元。市本级、肇东污水处理厂建成投入运行，安达、明水、庆安城市污水处理厂主体工程过半，其他县也在筹建。房地产业有序推进。以棚改为重点，全市房地产开发面积413万平方米，总投资50亿元以上。建筑及房地产业为全市财政贡献税收4亿多元，占整个地税收入的近三分之一，同时拉动了几十个相关产业发展。公共事业投资增幅较大。教育、医疗、卫生、文化、体育、环保等投资同比增加30%。

——现代农业和新农村建设又登新台阶。农业增加值实现213亿元,同比增长11.7%。大力实施粮食产能工程，建设重点农业基础项目和高产攻关田项目39处，组建千万元以上农机合作社17个，总投资33亿元。“肇兰青”10万亩玉米高产攻关田项目得到胡锦涛总书记肯定，“庆北绥”200万亩水田现代示范区项目在全省和全国现场会上推广。在多种自然灾害情况下，粮食总产实现223.9亿斤，再创历史新高，农村人均贡献粮食2.8吨，被《人民日报》以绥化市筑起“绿色大粮仓”为题头版头条报道。全国产粮大县增加到6个，肇东再获全国产粮大县标兵殊荣，海伦、北林、望奎、青冈、庆安被授予全国产粮大县。把畜牧业作为农业结构调整的重点，以生猪、奶牛两大工程为牵动，肉、奶、蛋产量大幅提升，畜牧业增加值实现80亿元。用经济手段推进生态林业发展，完成人工造林41.8万亩，居全省首位。森防实现全年无火灾，支援域外防火受到省政府表扬。整合资源、创新机制，持续增加新农村建设投入，村镇建设总投资42.76亿元，增长1.7倍。投入改造资金30亿元，又使5万户农民告别了泥草房。庆安、肇东、明水、北林、绥棱等地通过土地整理，整屯搬迁，异地改建，既提高了资金使用效益，又集约利用了土地。13座中小型水库除险加固工程开工建设，肇东段松干堤防全面竣工。全市又有17万农民吃上了安全水。绥棱县加大改造投入，农村自来水入户率达80%。全市农村道路硬质化、休闲公园化、住房砖瓦化、服务社区化进程加快，村级文化活动场所建设走在全省前列。加强对农民工就业扶持，全市内转外输劳动力108万人，劳务收入60亿元。

——工业经济速度、质效同步提升。大打科技长入、税费减免、金融支持、环境保障组合拳，工业经济不降反升。规模以上工业完成产值213亿元、增加值62.8亿元、销售收入210亿元、利税33亿元，分别增长18.9%、24.2%、20.6%和34.8%，均超全省平均增幅。强化重点行业和骨干企业拉动。帮助协调贷款15亿元，争取新型工业化专项、技术改造和贷款贴息等资金3663万元，协调国储粮和陈化粮110万吨。全市食品行业对工业增长的贡献率超过50%，工业近7成的增加值和8成以上的利税来自骨干企业。肇东中粮、伊利乳业、安达贝因美、青冈龙凤、望奎双汇北大荒、庆安鑫利达、明水三精制药等企业的平均增速都在30%以上。肇东大庄园二期、望奎成达肉鸡、北林段氏面业、海伦九鼎酵母等新上企业增势明显。加强园区载体建设。全市17个工业集聚区基础建设投入2.4亿元，新入驻重点项目39个，项目聚集效应愈加显现。哈大齐工业走廊肇东、安达项目区建设成效显著，辐射示范效应迅速扩大，又新引进大项目18个，累计入驻大项目70个；青冈工贸集中区起点高、气魄大，发展前景喜人；望奎西郊工业园、北林东富开发区、海伦工业开发区、庆安农产品加工区、绥棱九江工业园、明水格林和三精工业园、兰西亚麻工业园等基础设施日趋完善，承载能力进一步增强。全市培育了3个国家级中小企业创业基地，3个省级以上中小企业服务平台。加强科技创新和产品开发。新认定国家级高新技术企业5户，新建产学研联合体5个，全省唯一的牛奶检测中心落户安达。肇东中粮、青冈龙凤、兰西朝阳3个省级企业技术中心作用日益加强。全市工业开发新产品50多种，获省级优秀新产品奖2个。加强环境保护，全面完成年初确定的节能减排目标。

——商贸流通和现代服务业迈出新步伐。稳外需、扩内需，努力放大新的增长点。进出口贸易企稳回升。实现进出口贸易额7345万美元，虽然同比下降6.5%，但小于全省降幅23个百分点，其中高新技术产品和机电产品出口额增长47%，对俄贸易超额完成任务；实际利用外资3774万美元，增长4%。万村千乡市场工程和家电汽车下乡工程顺利推进。改造提升县级配送中心10个，新建改建农家店600个，基本实现村屯全覆盖；销售汽车、家电下乡产品近11.5万台件，兑现补贴6700多万元，有力地扩大了农村消费。现代物流业加快发展。全市各类交易市场、专业批发市场发展到215个。海伦大豆批发市场形成了买龙江、卖全国的集散效应；绥化申禄达国际家居建材城、电子商贸城、望奎农资批发市场建成营业；青冈工贸物流园、北林生资连锁配送中心建设进展顺利；全市新建专业物流公司30多家，并在全省率先组建了区域性粮食集团。物流经济对全市财政贡献率增长10%，安达市仅第三方物流就对财政贡献税收1.2亿元。金融服务水平提高。全市金融机构累计投放贷款250亿元，同比增加82亿元，增幅达49%，其中信用社投放贷款83亿元。哈尔滨银行在绥化设立一级分行，大庆商行落户肇东。兰西朝阳公司在美国柜台公示板上市，填补了我市上市公司的空白。全市成功申报小额贷款公司10家，肇东、兰西、青冈小额贷款公司已开业运营。建设银行发起的肇东村镇银行已纳入省规划。全市保险业实现保费收入14.9亿元，同比增长21.14%。全市金融业在支持地方经济发展的同时，为财政贡献税收1.5亿元。生态休闲、农业观光、民俗文化等地方特色旅游实现长足发展，旅游业收入实现5.6亿元，增长17%。社区服务业渐入佳境，在增加收入、带动就业方面发挥了重要作用。全市第三产业增加值实现247亿元，占GDP比重提高到40.7%。非公经济进一步壮大，实现增加值296.9亿元，增长21.2%；实现税金19.1亿元，增长20.1%，占全口径财政收入48.5%。

——市本级经济社会发展全面提速。经济总量、质量持续提高。完成财政总收入13.5亿元，增长13.3%，自身可用财力达10亿元，均创历史新高。北大荒米娃、索康乳业、新安工业硅、宝利多晶硅、申禄达大市场等8个项目投产运营，为优化本级产业结构和经济持续快速增长奠定了良好基础。招商引资成效显著。新引进亿元以上项目16个，总投资超过62亿元，是引进项目最多的一年。深圳中晶太阳能电池板、中粮30万吨稻谷加工、郑州白象方便面、浙江海运集团水泥、吉林中豆、大连石头造纸、北京首龙再生资源加工、美国细胞酶、南京润恒物流等项目不论是投资规模，还是发展前景，都提升到了一个新层次。硅基材料产业被列入全省新材料产业发展规划，必将有力促进该产业发展。开发区基础设施建设加强。投资1.8亿元的22万千伏变电所基本建成，铁路专用线建设筹备工作正在抓紧进行。城市开发建设继续推进。投入3000多万元，建设主次干道和巷路33条，累计修路已超过200条，新增排水管线9712延长米，新增路灯4000多盏。投入5000万元，开展了“十百千”创绿行动，新增绿化面积30多万平方米，被评为全省城市园林绿化先进市。中盟热电实现满负荷供热，新增集中供热面积120多万平方米。投资1.6亿元的污水处理项目建成。制定出台了促进市区棚户区改造和商品房销售的激励政策，推动房地产开工面积达116万平方米。二、三级房地产市场交易活跃，交易额达8亿元。快速发展的房地产业，既拉动了住房消费，又在很大程度上抑制了房价上涨，同时又为当年财政集聚资金5亿多元，仅地产净收益就贡献了2.69亿元，纯增1亿多元。各项惠民工程如期完成。拆迁棚户区40万平方米，开工建设75万平方米，均超计划1倍，又有6000多户居民陆续搬进新楼房。配建廉租房879套4.4万平方米，为5712户住房困难户发放补贴1840万元。棚户区改造累计政策让利百姓资金达6亿多元。社区建设成效显著。2个1000平方米、3个400平方米、5个300平方米的社区办公和活动场所建成。机关事业单位第二步津补贴全部发放完毕，人均实际增资350元。科技馆正式开馆。五星级宾馆和中国绿色产品特供中心开工建设。地下商业街如期开业，增加就业岗位1300多个。新区实验学校建设开始启动。城市管理加强。交管数字化监控系统投入使用，“三优”文明城创建取得积极成效，城市环境进一步改善。

——民生和社会事业取得扎实成效。在财政支出压力有增无减的情况下，克服困难，兑现承诺，仅向上争取和投入重点民生资金26亿多元，加上地方财政投入及政策性投入，总投入超过40亿元，是投入力度最大的一年。社会保障能力不断增强。在就业保障上，全市城镇新增就业5.9万人，开发公益性岗位5498个，城镇登记失业率控制在4.2%以内。开展“三支一扶”和就业见习活动，高校毕业生就业率达到83.5%。为1969人发放小额担保贷款4400万元，超额完成了省定目标。在住房保障上，投资40多亿元，新增棚改建筑面积345万平方米，投资30亿元，改造泥草房5.4万户462万平方米。配建廉租房33万平方米，又有6613户城市低收入家庭住上廉租房，圆了多年的住楼梦，还有31321户城市低收入家庭享受到了廉租住房补贴。全市对2500户农村倒危房进行了改造重建，望奎县通过集中改建，解决了900多农村住房困难户的安居问题。大力度的棚户区和泥草房改造惠及城乡居民11.2万户，是全市投入最大的民生项目。绥棱、青冈等地把棚改与打造城市文化带和功能区相结合，更有力地扩大了棚改效果。全市新增城市集中供热面积270万平方米，超计划80%。在养老保障上，投资5135万元，新建县级中心敬老院6所，全市中心敬老院发展到21所，居全省首位，海伦、绥棱、青冈五保村建设初具规模。把2.79万名“五七工”、“家属工”纳入养老保险，实现了老有所养。全市为14.17万企业离退休人员发放养老金13.59亿元。在弱势群体保障上，城乡纳入低保人员达46.6万人，增加4万人，发放救助资金6.7亿元，向农村受灾群众提供生活补助5533万元，发放口粮6700余吨。价调基金救助困难群体规模进一步扩大。大力开展扶残助残活动，残疾人事业迈入全省先进行列。整村扶贫、千会带万户和万名妇女解困工程取得明显成效。在医疗保障上，新建续建扩建医疗机构32所，“三网四化”建设稳步推进。新农合参合率达到93.8%，补偿金额超2亿元。城市“双低”人群医疗救助8.2万人，救助资金780万元。全面启动城镇居民医疗保险，参保人数扩展到92.98万人,位列全省第三。甲型H1N1流感防控有力，全市无暴发性疫情发生。农村中医工作进入全国先进。教育水平不断提升。“教学质量升级年”活动取得实效，实施直属学校监管包保责任制，促进了办学水平提高，得到了教育部肯定。投入2.6亿元，实施校舍安全工程、危房改造和标准化学校建设统筹推进，标准化学校建设规模超计划完成50%。社区建设跨越发展。投入2.07亿元，建成1000平方米县级社区服务中心15个，500平方米街道社区服务中心36个，150—300平方米社区服务站76个，分别是计划的2.1倍、3.6倍和1.9倍，建成服务网点3593个。全市161个社区的办公用房全部解决。肇东城市社区建设经验在全省推广，庆安农村社区建设经验在全省和全国推广。科技、文化、体育、广电、人口、土地、环境建设等事业实现新突破。实施省级以上科技项目62项，投入资金达1.5亿元，科技特派员工作受到全国表彰。望奎满族风情园、庆安文化艺术中心、明水文化中心等一批特色鲜明的文化场馆相继落成；新建标准化乡镇文化站36所、农村书屋300个。竞技体育加强，青冈籍运动员李艳凤荣获第十一届全运会女子铁饼冠军，实现了我市全运会史上金牌零的突破。有线电视实现“村村通”，数字电视提前开通。人口计生工作扎实有效，自然增长率控制在3.26‰，计生站建设工作完成了代表省接受国家“十一五”中期评估任务。土地保障保护工作加强，在千方百计保证重点项目建设用地的同时，地产综合收益再创新高达到9.29亿元，耕地保护工作名列全省前茅，新增耕地面积994公顷。城乡生态环境改善。城市公园、广场建设和绿化、亮化、美化力度进一步加大。绥棱县城市公园广场面积近100万平方米，人均达到17.5平方米，走在全市前列。全市新建省级环境优美乡镇8个，生态乡镇10个，生态文明村96个。社会保持和谐稳定。重塑警风、重振警威，公安系统“百日整顿”取得可喜成效,打击力度加大，百姓满意度和安全感增强，“百日整顿”经验得到上级和全市各界充分肯定。深入开展“信访积案化解年”活动，狠抓源头治访，多项工作经验在全省推广，信访稳定工作多次受到省委省政府表扬。价格监管和价格服务成效显著，价格杠杆作用明显，为全国、全省提供了经验。强化市场管理，食品、药品、农畜产品等市场专项整顿取得实效。加强安全保障，全年无重特大生产安全和交通事故，连续十年被省政府评为安全生产责任制优秀单位。

此外，全市妇女儿童、审计监察、民族宗教、外事侨务、老龄工作、新闻出版、防震减灾、社会科学、统计咨询、史志档案、法制、人防、双拥等各项社会事业也都取得了新成绩。

回顾一年来的工作，我们之所以能够化解金融危机的不利影响，顶住压力，战胜困难，取得可喜成绩，主要得益于省委、省政府和市委正确领导；得益于人大、政协、中省直部门和社会各界戮力同心、鼎力相助、共克时艰；得益于我们准确把握形势，积极应对挑战，实施一系列符合实际的调控举措；得益于我们不断加强政府自身建设，解放思想，弘扬实干精神，打造阳光政府，提高办事效率；得益于我们始终坚持民生为本、发展为先、责任为重，精诚团结，勤政廉政，以过硬的作风抓推进、抓落实。在此，我代表市政府向一年来为绥化发展做出贡献的各级组织、各界朋友和各位同志，致以最诚挚的感谢和最崇高的敬意！

在看到成绩的同时，我们也清醒地意识到，经济社会发展中的困难和问题还不少。一是财政收入总量小，与各项刚性支出增加的要求比，压力还比较大。二是招商引资和项目建设进展不平衡。三是由于基础较差，就业、医疗、养老等民生工作任务还很艰巨。四是政府职能转换还不到位，个别部门还存在效率不高、环境不优、落实不力的问题。五是城市管理还有待加强。对于这些问题，我们要以高度负责的精神认真加以解决，不辜负全市人民的期望。

二、2010年政府工作的主要任务

各位代表，2010年是加快发展的关键一年。机遇挑战同在，动力压力并存。一是国际经济缓慢复苏，国内经济企稳向好；国家继续实施积极的财政政策、适度宽松的货币政策，继续加大对民生、对三农、对社会事业的投入，尤其是加强对欠发达地区的支持，加强对基层政府的财力保障；全省实施八大经济区战略坚定不移，并陆续出台六大新兴产业发展规划。如何乘势而上，加快发展，我们面临着更多的机遇。二是后金融危机时期，金融危机已演变成新一轮的发展竞争，倒逼机制催生整个世界经济，进入利益大挪移、结构大调整、产业大整合的新阶段；转变发展方式也由此上升为国家的重大宏观战略；顺应大势，国内许多发达地区超前进行了结构升级、经济转型；省内一些兄弟地市也在保增长中积累了更大的发展后劲。相比之下，我市结构调整相对滞后，发展相对不足，在后发优势上劣势更加凸显。如何好中求快、更好更快，我们面临着更大的挑战。三是去年以来，国家先后批复了9个区域发展规划，我省实施了八大经济区规划，标志着区域协作、一体化发展，已成为当今时代经济发展的主流；而我市突破城乡之间、县域之间和同类产业之间的“分割”束缚，实现统筹发展还没有很好的破题；同时金融危机对我市部分行业的影响还很大，经济回升的动力和下行的压力同时存在；加之大民生、大保障的高标准、新要求,深化改革的必要成本投入，结构调整的必要财力支撑，与我市“吃饭财政”的实情交织在一起，矛盾显得格外突出。如何履行科学发展、执政富民的神圣职责，我们面临着更重的考验。

新的一年，环境喜人，形势逼人。面对不进则退、慢进也退，别人“快走”、我们必须“快跑”的现实处境，我们必须坚定不移地加快发展。切实在发展中调结构，在优化结构中谋发展，在又好又快、更好更快上求突破；必须卓有成效地壮大财力。注重争取“上援”，更加注重“自主创收”，大力推进吃饭财政向富强财政转变，切实增强发展的调控力、民生的保障力；必须有的放矢地深化改革。聚焦重点，主攻难点，调整体制，优化机制，为推进发展、促进繁荣提供强有力的制度支持；必须不遗余力地改善民生。无论面临的困难多重，保障民生的力度不减；无论遇到的压力多大，改善民生的投入要保，强力推进民生事业晋档升位。2010年，政府工作要深入贯彻落实市委二届四次全委扩大会议精神，以科学发展观为统领，以“两区”建设为抓手，以大事大项为牵动，坚持发展至上、责任为重、民生为本、一切唯实的原则，加快发展保增长，持续发展调结构，创新发展促改革，全面发展惠民生，推动全市经济社会在跨越发展上迈出更大步伐。主要预期目标是：地区生产总值增长11%以上；规模以上工业增加值增长15%以上；固定资产投资增长35%以上；社会消费品零售总额增长15%以上；外贸进出口总额增长15%以上；财政一般预算收入增长11%以上；非公经济增加值增长16%以上；万元GDP综合能耗下降3%；城镇居民人均可支配收入和农民人均纯收入分别增长8%和6%以上；人口自然增长率控制在5‰以内。

具体抓好八项重点工作:

（一）全力扩大固定资产投资，进一步增强发展后劲。抢抓机遇，自我加压，大力推进固定资产投资又好又快增长。千方百计对上争取投资。全面把握好国家和省的扩内需项目，对去年已争取到的11.28亿元国投项目资金，做好匹配，确保高标准建设，以利争取更大支持。进一步选准拟争项目。重点把握“千亿斤粮食产能”规划、强化“三农”等事关国计民生的大局争取支持；把握加快中小城镇发展和继续加大对民生及社会事业的投入争取支持；把握注重在建项目后续配套、节能环保的大导向争取支持；把握对欠发达地区补欠账、促均衡争取支持。进一步增强工作的主动性、科学性，力求对上争取更多的预算内投资项目。努力争上新项目拉动投资。继续加大招商力度。着眼于做大做强现有主导产业，进一步加强产业链招商和以商招商，巩固低端，做强中端，抢占高端；着眼于大力培育新兴产业，加强与省内外科研院所的技术合作，加强与高科技企业，特别是国内外大集团合作，引进一批科技含量较高的吃配项目，加快使部分新兴产业成为支柱产业；着眼于积极抢抓产业转移引资上项，充分利用金融危机引发市场重新“洗牌”，加强与发达地区间合作，努力成建制的引进一批转移项目。每个县市要确保引进投资超亿元项目2个以上，超5000万项目3个以上，到位资金增长20%以上。扩大开放吸引民间投资。按照“非禁即入”的原则，进一步开放市场，鼓励支持域内外各种民营资本投向国家鼓励和“不禁止”的各个领域。加大城镇开发力度，用最开明的政策、最优良的环境、最优质的服务吸引投资者参与城市开发，特别是小城镇开发。要以房地产开发的强劲动力扩大城镇固定资产投资规模。发挥政策和财政资金的激励引导作用，推动民间资本更多的参与城镇交通、卫生保洁、美化绿化和“三供两治”等基础项目建设。拓宽投资领域，助推民间资本参与农业产业化和现代农业的开发建设。把握集约发展科学引导投资。加强市本级经济开发区，哈大齐工业走廊肇东、安达两大项目区和其他工业集中区的配套建设，增强辐射能力，扩大集聚效应。学习借鉴海林等地经验，创新项目区建设方式，借力扩张，组团打造，引导固定资产投资向壮大产业龙头、培育产业集群上发展。打破行政区划界线，鼓励支持县际、城乡、乡镇等之间联手创建产业带、加工区和农产品生产基地，发展飞地经济，广泛吸引各路资金投资兴业。倍加珍惜土地资源，严格掌控投资密度，为固定资产投资持续增长提供必要条件。

（二）大力发展现代农业,扎实推进社会主义新农村建设。以抓好“十大工程”为着力点，深入落实农业综合开发试验区规划，发挥比较优势，积极推进现代化大农业建设，统筹城乡发展，全面提高新农村建设水平。全力抓好以高产创建为突破口的粮食生产。深入实施240亿斤粮食产能规划，强基础、重科技、提效益、攻单产，确保粮食产量稳定在220亿斤左右。加大玉米、水稻、大豆高产攻关推进力度，整合各类资金，持续增加投入，落实好全市32个重点攻关核心区，牵动高产创建突破800万亩。加强以水利、农机、生态、培肥地力为重点的基础建设。全市新增抗旱大井2000眼，全面完成13座中小型水库消险加固、永安渠首二期改造和肇东段松干堤防建设任务，广用地下水、拦蓄江河水，逐步摆脱雨养农业束缚；发展壮大农机合作社,加强场县共建，每个县（市、区）都要与农垦联合建立大型农机合作社，全市大型农机总动力突破300万马力；坚决落实省委、省政府三年大造林的决策部署，确保完成人工造林33.5万亩。继续实施“沃土工程”和黑土区保护工程，抓好深松整地和秸秆根茬还田、过腹还田，深松整地1500万亩以上，新增黑土区治理面积30万亩以上。强化科技对高产创建的支撑，加强各类良种技术、先进栽培技术、施肥技术、节水技术、生物技术、纳米技术和农防林断根覆膜技术的推广应用，让先进科技广泛长入农业。全力抓好以增加农民收入为核心的结构调整。紧紧围绕促农增效、促农增收，以抓好寒地黑土品牌升级为牵动，深化农业结构调整。种植业往绿色有机上调，进一步建立完善寒地黑土产品生产标准，着力抓好标准的使用和推广，扩大绿色有机粮食作物和高效经济作物种植，放大价格及收入的倍增效应。全市绿色作物种植稳定在1500万亩以上，有机食品种植15万亩以上，种植业标准化率和优良品种率提高到91%和92%。推广肇东、青冈等地经验，大力发展城市和乡镇周边的棚室瓜果和蔬菜生产，引带示范，扩大规模，同时积极发展庭院经济。加快市级绿色食品检测中心建设，争取早日立项。养殖业往规模优质上调，深入抓好150万吨奶和1300万头生猪发展规划的落实，大力搞好规模化、标准化养殖。实施“百千万”工程，百头以上奶牛养殖场区、千头以上生猪养殖场区、万只以上养鸡场区分别发展到50个、300个、100个以上。搞好动物防疫，加强质量安全监管，确保不发生重大疫情和安全事故。全市畜牧业增加值要突破90亿元。农业组织方式往产业合作、专业协作上调，大力发展各类产业化经营组织和农民专业合作组织，发展各类特色种养村屯和专业村屯，引导农民靠合作致富，靠有组织的分工分业致富。全市各类农民专业合作组织发展到5000个。高度重视解决好龙头企业与基地农民的利益连接问题，促进农业产业化健康发展。有组织地搞好劳务输出，在巩固数量的基础上，加强就业技能培训，打造更多的劳务品牌，大幅度增加农民的工资性收入。全力抓好以中心村镇为重点的新农村建设。把握农村城镇化的发展趋势，科学规划设计新农村的建设蓝图。按照壮大中心村、撤并过小村屯、发展中心镇的原则，进一步把中心村镇的建设突出出来，防止和避免盲目建设。推广庆安、肇东、明水、北林、绥棱等地的做法，结合泥草房改造，整体迁移一些过小村屯，扩大中心村规模。充分发挥小城镇在新农村建设中的龙头作用，抓住中央扶持中小城镇发展的历史机遇，力争在小城镇基础设施建设和功能完善上取得突破。继续加强农村路、水、电、医、教、能等公共事业建设。建设农村公路1500公里；新增安全饮水工程300处，解决15万人安全饮水问题；新增大型沼气工程3处以上，抓好秸秆汽化固化燃料的开发推广；多渠道培训教育农民，加快提高农民素质。实施环境整治工程，搞好“三清、四改、五化”和“四旁绿化”。

（三）努力提高工业运行质效，增强发展的牵动力。着力优化工业结构，扩充总量、提高效益。在巩固做强食品、化工、医药、机电、纺织等现有主导产业基础上，大力发展新兴产业，拓宽发展空间。进一步做大做强现有企业。采取滚动招商、联大靠强、技术改造、工艺创新等措施，促进现有企业强筋壮骨，发展壮大。借鉴肇东、安达、望奎、青冈、明水、海伦等地做法，以商招商，滚动发展，借力大企业、大品牌，着力在配套建设、精深加工、梯次增值上寻求突破。加强对30户重点骨干企业的跟踪服务，重点扶持一批潜力较大的企业加快成长，尽快把市本级新安、保利、昊天、肇东宝迪、大庄园、安达贝因美、亚泰、望奎双汇北大荒、青冈龙凤、明水格林、庆安鑫利达、北林大众、海伦南华、兰西朝阳、绥棱嵩天等一批企业打造成产值30亿、50亿、100亿的重量级企业。加强区域间分工协作，积极引导关联度较强的重点行业、企业联合重组，集中打造有实力的企业集团。加大技改力度，实施低成本扩张，从资金和人才上支持企业对新技术、新工艺、新产品的引进和开发，帮助有条件的企业成立技术中心，提高研发实力。年内每个县（市.区）都要设立中小企业发展基金，加大对成长型中小企业的扶持，着力培育壮大“小规模、大群体”的产业集群。同时抓好新上企业的投产达效，研究好停产半停产企业的转型措施，杜绝摆设项目和半截子工程。加大产业结构调整力度。抓住国家支持鼓励发展战略性新兴产业的机遇，瞄准全省提出的六大战略性新兴产业，积极引进，主动配套。进一步在硅基材料、化工材料、新型建材、生物发电、风力发电、生物医药、现代中药、生物农业等有一定基础的方面，加大力度，率先发展，争取在发展战略性新兴产业上有所作为，有所突破。加大对新上项目的调控力度。对生物发电项目，要考虑原材料半径，防止一哄而上。对资源加工转化率较高的玉米、水稻、乳品、肉类、甜菜、亚麻等加工业避免低水平重复建设，避免对区域资源的无序争夺，严格控制初加工项目进入。围绕产业化经营，培育扶持“一乡一业、一村一品”的块状经济发展。扎实推进节能减排。大力发展低碳经济、绿色经济，努力实现绿色增长。抓好重点耗能企业的降耗监管，对国家重点监控的肇东中粮生化和省考核的11户重点耗能企业，实施能源年度审计和节能规划的审核工作，确保完成节能降耗目标。对已建的50个工业企业污染治理设施要确保正常运行。提高招商引资门槛，严把项目准入关，严格限制高投入、低产出、高消耗、低收益、污染重的项目进入，从源头上抓好节能减排。

（四）搞活商贸流通，推动第三产业成为加快发展的增长极。大力促进商贸经济扩总量、优结构，推动整个第三产业加速成长为支柱产业。努力扩大内外贸总量。围绕扩大内需搞活传统商贸业。大力开展形式多样的促销活动，聚焦扩大更多消费热点。积极引进国内外大型超市和商场，满足多层次的消费需求。继续推进“家电汽车摩托车下乡”和“汽车以旧换新”工程，优化补贴方式，进一步激活农村消费。深入挖掘进出口贸易潜力，重点开拓俄、日、韩市场。用足用好中小企业国际市场开拓资金、外贸发展资金、高新技术和机电产品进出口贴息等政策性资金，积极培育壮大外贸出口群体，使有进出口实绩的企业发展到40家以上，同时努力变间接出口为直接出口。调整优化出口产品结构，提升产品内在质量，力推机电、高新技术等高附加值产品的出口份额增长20%以上，对俄出口增长10%以上。大力培育发展大型骨干市场。发展大市场，推进大流通，重点建设完善一批规模大、集散功能和辐射力强的的大型专业市场。加强对申禄达国际家居建材城、欧亚达农机大市场、海伦大豆批发市场等大型骨干市场的扶持，增强内外吸引力、扩张力。积极推进兰西亚麻市场升级改造，加快青冈工贸物流园建设，提升望奎大龙商贸城功能。培育发展大牲畜交易市场、生资市场和建材批发市场。继续抓好“万村千乡市场工程”，力争再改造和新建农家店300家以上，综合配送比重达到70%以上。规范各类市场经营秩序，努力打造良好的市场环境。优先发展现代服务业。大力发展现代物流业，加大对现有物流企业的扶持力度，注重发展第三方物流，下力量引进大型物流项目，加快壮大物流群体。大力发展新型业态。积极发展连锁配送、直销经营、电子商务、特许经营等现代流通方式；支持金融保险、拍卖典当、资产评估、公证鉴定、会计审计等中介机构加快发展。积极发展以社区为主体的居家养老、扶残助孤、家政服务产业，拓宽内容，扩大网点，强化管理，并使其向连锁化方向发展。认真抓好房地产开发和旅游业发展。充分发挥房地产业拉动投资和扩大消费的积极作用。全市房地产开发力争突破500万平方米，总投资70亿元以上。继续以观光农业和生态旅游开发为重点，抓好具有地方特色的乡村游、田园游、农家游、观光游、民俗游、冰雪游、休闲游、怀旧游等，创新融资手段，靠政府引导和市场驱动，推进精品景区建设，借势造势，提高我市旅游业的知名度和影响力。

（五）推进重点领域改革，进一步增强发展活力。围绕破解发展难题、优化发展环境，从制度安排入手，大力推进体制机制创新。继续深化农村改革。重点抓好以土地规模经营为核心的土地流转，积极发展土地合作社。大力度推进乡镇农技推广和兽医体制改革，上半年要改革到位。从体制机制上推动乡村两级职能转变，大力发展乡镇经济，完善农村“三资”代理，努力壮大乡村两级财源。继续深化企业改革。完善国有和国有参股、控股企业的法人治理结构，进一步深化国有粮食购销企业改革。支持企业并购重组，做大做强。放胆放手发展民营经济，进一步营造百姓兴家业、能人办企业、干部创事业的全民创业氛围。完善政策调控，运用各种经济杠杆尤其是土地、环保等关键要素，促进形成有利于加快企业转变发展方式的体制机制和利益导向，推动自主创新，推进产业结构调整。积极稳妥地搞好政府机构改革。按照“整合职能相近机构，清理规范议事协调机构，将事业单位行政职能划归行政主管部门”的大原则，精心制定“三定”方案，科学整合相关机构。深化干部人事制度改革，鼓励支持有条件的机关干部离岗创业。深化行政审批改革，进一步转变政府职能，提高办事效率。搞好事业单位分配制度改革。适时启动绩效工资制度改革，以贡献定报酬，激发干部职工干事创业的积极性。对职能严重弱化，无力组织创收的事业单位，条件成熟的，开展事业单位改革试点，条件不成熟的，在保持人员身份不变的前提下，实行人员分流，减少人力、财力浪费。搞好医药卫生体制改革。积极探索公立医院改革和实行基本药物制度的有效模式，加快推进医药分离。抓紧搞好基层医疗机构药品零差率试点。创新城市医保和新农合工作机制，完善分级就医就诊制度，完善药费核销程序，在医疗便民、利民、惠民上实现新突破。推进其他配套改革。按照国家和省的总体部署，及时开展资源价格和财税体制改革、环保收费改革、动拆迁制度改革、养老保险改革。充分考虑市情实际，适时启动公务用车改革、城管体制改革和经济开发区、工业集中区管理体制改革。

（六）认真做好财政金融工作，提高发展的保障力。采取有效措施，推进财政、金融事业与经济社会发展形成更紧密的良性互动机制。大力强化财税建设。更加注重把发展经济和社会事业与增加财政税收有机结合，把财政增收作为衡量发展质量、检验结构调整的重要标尺，有的放矢地加大创税农业、创税工业、创税服务业的建设力度，优化财税结构。注重发挥财政资金“四两拨千斤”作用，扶持重点企业提高创税能力，引导民间资金注入优势产业，激活社会沉淀资本。积极创造条件，大力发展总部经济、结算经济。继续加大税费征管，确保应收尽收，确保财政收入较快增长。努力优化支出结构。严格控制一般性支出。进一步加大对民生事业、对深化改革、对维稳创安的支持力度，集中财力履行好公共财政职能。着力完善投融资体系。积极协调银行扩大贷款规模，创新金融产品，保障重点项目建设资金需要。积极争取国有商业银行在我市一些县恢复分支机构，鼓励支持增设服务网点，并积极引导境内外金融机构在我市设立机构。要通过政府资本注入、风险补偿、政策扶持等措施，加快信用担保体系建设。市县两级都要拿出一部分财政资金用于支持中小企业融资平台建设，有效缓解中小企业融资难问题。加快推进市城投公司建设，扩大直接融资规模。支持信用社扩大服务领域。加快村镇银行、小额贷款公司发展。积极改善金融环境。强化对信用资金的运行监控，探索建立“黑名单”、“红名单”制度，加大失信惩戒力度；建立对金融案件的集中审结制度，提高司法效率，从重从快打击各种恶意逃废债行为，构建经济与金融和谐发展格局。

（七）加快市本级发展，全面提升综合实力。深入实施城市开发和项目牵动战略，着眼于打牢基础、加快发展，工作重心由城市开发与项目建设并重向更加注重项目建设转变，向更加注重管理城市和完善城市配套功能转变，力争在经济总量、产业发展、精细化管理上有一个大突破，同时继续加大改善和保障民生的力度。预期目标是：规模以上工业增加值增长15%以上，全口径财政收入增长11%以上,一般预算收入增长11%以上。开发区产值、增加值、利税三项指标增幅达到20%以上。全力抓好以开发区为重点的大项目建设。围绕壮大农副产品精深加工、硅基材料和物流产业规模，加快把开发区打造成牵动市本级发展的龙头。在巩固发展现有企业基础上，加大已签约项目的落实力度。重点抓好郑州白象方便面、中粮30万吨稻谷加工、美国细胞酶、吉林中豆食品、大连信德石头造纸、浙江海运水泥、南京润恒物流等14个重点项目的开工建设。抓紧落实土地，确保早日建成投产。加快硅基产业发展。抓住省里把绥化列入发展硅基新材料产业规划的机遇，乘势而上，向深度加工拓展，加速建成以工业硅为基础，以有机硅和多晶硅为核心，以有机硅深加工和光伏产业为延伸，上下游配套发展的硅基新材料产业基地。促进已经入区的新安、宝利两户企业尽快达产达效，抓好深圳中晶公司年产50万千瓦太阳能硅电池板项目、保定拓日太阳能LED光电产业基地项目的入区建设，壮大硅基产业整体阵容。力争通过3—5年努力，把硅基产业做成百亿产值以上规模。配套跟进基础设施建设。抓好22万伏输变电站建设，确保尽快投入使用。积极抓好铁路专用线建设，争取早日开工。推进开发区向多功能区方向发展，抓好新扩容区块道路、给排水、供电等基础建设，加快生活区建设，切实提高承载能力。抓好城市扩容工作，鼓励支持农村人口进城创业。继续搞好城市开发，配套完善城市功能。加强规划管理。坚持房地产开发、棚户区改造与城市基础设施建设相适应的原则，进一步搞好城市总体规划和土地利用规划的修编工作。突出做好棚改项目、大唐热电联产项目、康庄路周边发展项目的规划服务，并及时跟进市政基础设施建设。搞好15条城市主次干道巷道和燃气管道建设的规划设计与实施。加强对规划项目建设的跟踪管理，严格规划执法。继续加大房地产开发和土地市场运营力度。坚持棚改与开发并重，房地产新建续建面积200万平方米以上，新建100万平方米以上。同时抓好海融大厦、东城客运站、大世界家居广场等标志性工程建设。完善城区道路。重点抓好人和西街、绥望路等主次干路拓宽改造，主巷路全部实现硬覆盖。及时跟进给排水、路灯等配套设施。加强城区美化绿化。继续抓好中兴路精品街和康庄路、兴绥路绿色生态景观轴建设，提高城市品位。以中直路、新兴路、分区路等为重点，抓好街路绿化；以新建小区为重点，抓好庭院绿化；结合棚改预留相应的绿化空间，使城区绿化覆盖率达到32%，人均公共绿地面积达到6.1平方米。加强公园广场建设。抓好人民公园的改造提升，辟建水利排灌、挂面厂、清真寺等三个广场及植物园。加强城市管理。大力整治城市“三乱”，切实加强交通、市场秩序管理，探索城市保洁社会化、市场化运作方式，创造更加良好的城市环境。继续加大改善和保障民生力度。加速推进棚户区改造。在原有31个区块基础上，再新增连片拆迁区块15个，拆迁面积30万平方米以上，建筑面积60万平方米以上，配建廉租房2.8万平方米以上。抓好集中供热和天然气工程。对投资25亿元大唐热电联产、投资3亿元的中油天然气项目，争取尽早立项上马。加大社区建设力度。新建5个社区综合服务站，2个社区服务中心。同时，抓好儿童福利院、残疾人综合服务中心和新区九年制实验学校建设。推进市中等职业技术学校向高职升格，努力扩大招生规模。抓好市本级图书馆、文化剧场论证立项，争取纳入省政府投资项目。积极筹备办好“市三运会”等大型文体活动。

（八）抓好重点民生工程建设，推进社会事业全面发展。筹措更多的财力，投入更多的精力，全力推进重点民生工程。继续扩大就业再就业。广开就业渠道，深入实施创业带动就业战略。着力解决好高校毕业生、“4050”人员、零就业家庭成员和退伍军人的就业问题。鼓励支持农民工返乡创业。确保全市城镇新增就业5.5万人，下岗失业人员再就业5万人。不断增强社会保障能力。推进“五险”扩面，逐步纳入财政预算管理。重点做好城乡特困群体的养老保险工作，抓好新型农村养老保险试点。大力推进城镇居民医疗保险，参保人数突破100万人。积极做好农民工跨省参险的衔接工作。全面提高城乡低保标准，深入推进“双低”人员医疗救助保险工作。全面推进保障性住房建设。充分发挥政府主导、规划引导、政策引带作用，把保障性住房建设与完善城市功能、加快新农村建设、促进就业有机结合起来，全面提高“一棚一草”改造成效。全市完成棚户区拆迁面积241万平方米，配建廉租房37.2万平方米，发放廉租住房补贴1亿元；改造泥草房3.8万户、320万平方米；改造倒危房2100户、10.5万平方米。加快“三供两治”建设。9个县市的污水处理厂建设，要确保按计划如期交付使用，全面推进呼兰河污染综合治理项目。大力推进集中供热，新增供热面积270万平方米。加强城市供水管网改造，科学推进分户计量。继续推进教育优先发展。加快标准化学校建设，建立标准化校舍30所，完成中小学校舍加固改造98万平方米。大力推进中等职业教育发展，培养技能型人才1万人以上，农村劳动力转移和农民实用技术培训28万人次以上。强化医疗卫生保障。加快推进14个卫生基础设施续建项目。着力抓好新型农村合作医疗扩面工作，确保参合率达到98%。继续做好以甲型流感为重点的疫情防控工作，提高应急预警能力和处置能力。搞好社区服务和文体惠民工程建设。积极推进城乡社区和公共服务中心建设，逐步实现城乡社区建设全面覆盖、整体推进，城镇80%的社区办公用房实现自有化，打造30个农村精品社区。加强文体设施建设，加快城乡数字电视建设，推进城区数字电视整体平移。配套完善图书馆和文化剧场，新建24个乡镇文化站，建好100处全民健身工程和15处学校训练基地。深入开展扶贫解困。积极争取救灾资金，做好受灾群众冬春生活、生产救助。继续推进农村万名妇女解困、千会带万户、扶贫开发整村推进和城市特困职工解困工作，实现5万人脱贫。大力实施妇女儿童发展规划，推进重点难点问题有效解决。推进集中养老场所建设扩规模、提档次。充分发挥价格调节基金取之于民、用之于民的价格救助作用。认真做好残疾人就业、扶贫和康复工作，保障好残疾人合法权益。抓好人口计生、环境保护、土地资源基本国策的落实。继续抓好稳定低生育水平工作，统筹解决人口问题，进一步提高出生人口素质。认真落实环境保护各项政策要求，推进生态城市建设，加大生态文明乡镇、村屯建设力度。坚持保增长、促发展，保红线、促平衡，进一步提高土地工作为地方发展服务水平。抓好食品药品和农资市场监管。加强食品药品安全监管，完善责任体系，严厉打击制售伪劣食品、药品等违法行为。规范农资市场，严把市场准入，加强质量和价格监管，确保农民用上放心农资。抓好“平安绥化”建设。进一步加强社会治安综合治理，巩固扩大“百日整顿”成果，加大对黑恶势力犯罪、暴力犯罪、多发性侵财犯罪的打击力度，建立长效机制，推动打防管控工作整体上台阶。加大公安基础建设投入，努力提高装备水平。加强信访工作，确保信访总量、群体访量和越级非正常访量持续减少。坚持安全发展原则，严格执行“安全一票否决”，强化专项整治和日常监管，力保全年不发生重特大事故。加强价格监管，确保市场价格总体稳定。加强防震减灾管理，提高抵御灾害能力。继续抓好“双拥”工作，加强国防后备力量建设，加强民族宗教工作，促进社会和谐文明。

各位代表，完成2010年各项任务，是对各级政府抢抓机遇、应对挑战、践行科学发展能力的一次全面考验。我们必须强化政府自身建设。进一步解放思想、开拓创新。紧紧扭住加快发展，强化跑的能力、争的意识、引的本领，把握大局，主动用战略思维和跨区域合作的视野来聚合周边、乃至国际资源和市场，整合一切可以为我所用的条件，拓宽发展空间，提高发展质效。进一步强化服务、狠抓落实。切实把谋发展之道、惠民生之事、解企业之难作为首要任务，简化程序，提高效率，加强督查。对于定下来的事情，要全力以赴、敲钟问响，不抓则已、抓则必果，只有能行的义务，没有不行的理由。进一步发扬民主、依法行政。主动接受人大的依法监督、政协的民主监督、媒体的舆论监督和社会的全面监督，继续大力实施调研、咨询、听证、评估和集体讨论有机结合的重大事项决策机制，完善深化工程招投标、棚改、资金使用、人员录用等热点政务事项的公开办法，依法办事，照章理事。进一步从严治政、厉行节约。完善行政绩效考核，激发干部干事创业热情。认真贯彻落实党风廉政建设各项规定，强化对重点领域、重点部门、重点资金的行政监察和审计监督，规范权力行为。大力优化经济发展环境,切实纠正各种不正之风。继续深入开展节约节能活动，降低成本，杜绝浪费。我们各级政府班子及其组成人员，要始终牢记使命，不负重托，始终牢记百姓愿之所盼，就是政府责之所在，切实做到用好人民的权力为人民谋利，凝聚人民的智慧为人民造福！

各位代表！过去的成绩让我们站在了新的起点，百姓的期待又激励我们去实现新的跨越。让我们在市委的坚强领导下，坚定信心，迎难而上，众志成城，发愤图强，为推动全市经济社会跨越发展而努力奋斗！
